# Supplementary material for: Sparsely Connected Autoencoders: A Multi-Purpose Tool for Single Cell omics Analysis
Source: Int J Mol Sci. 2021 Nov 25;22(23):12755. doi: 10.3390/ijms222312755 (PMC8657975; doi:10.3390/ijms222312755)
Supplement: Supplementary file 1 [file ijms-22-12755-s001.zip › ijms-1418174-supplementary.pdf]

Supplementary Figures

Refined analysis of the single cell RNA-5c dataset transformed in TF metagenes by SCA analysis, see paragraph 2.4 on the main manuscript.

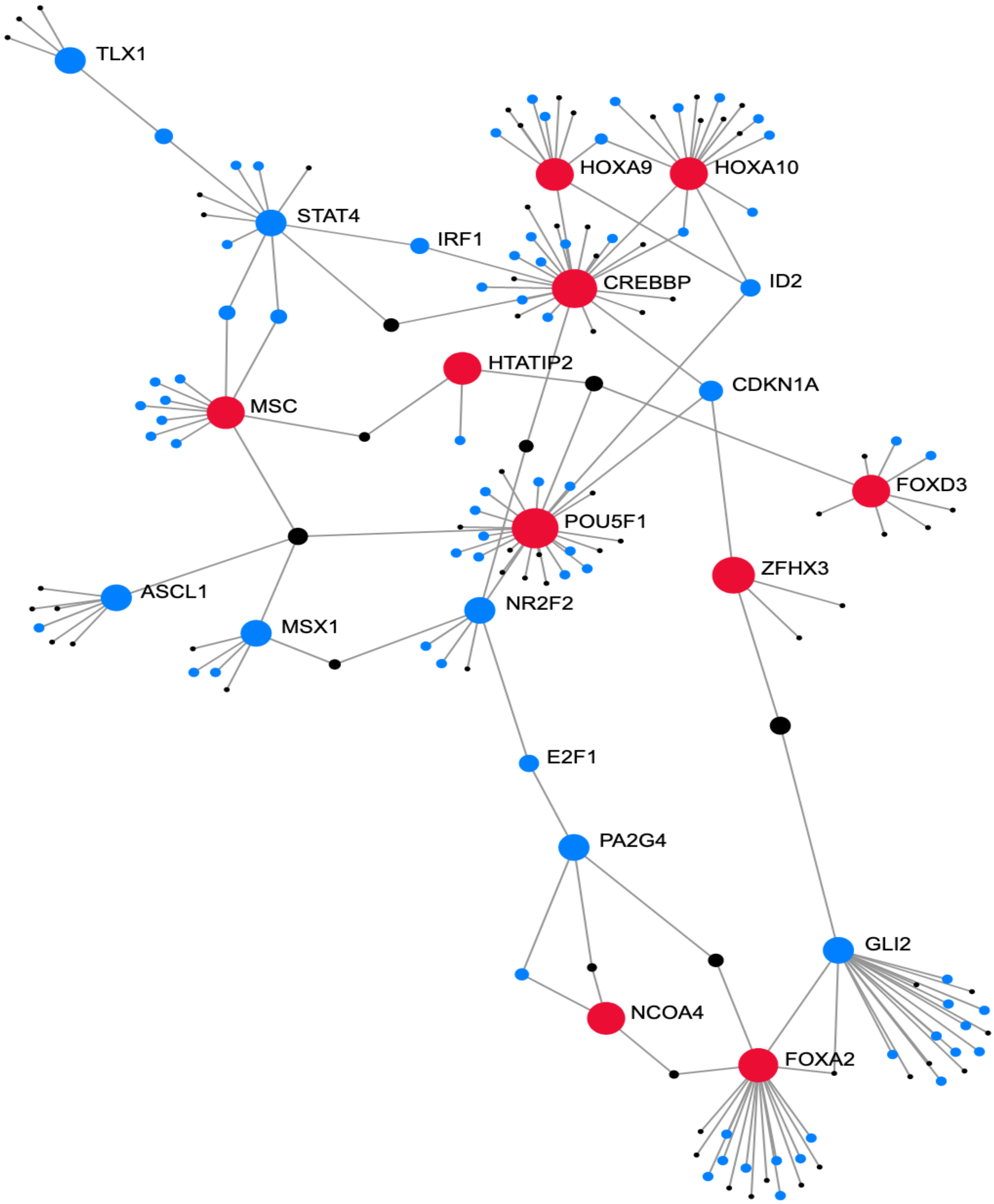

Figure S1: OmicsNet analysis of the transcription factors networks that can be generated using cluster 1 specific transcription factors. Large nodes with names refer to transcription factors, smaller nodes to target genes of the transcription factors. Blue color refers to transcription factors and target genes associated to “cell proliferation” GO Biological process term. In this figure are only represented TFs showing up-modulation in cluster 1 when compared with the other clusters.

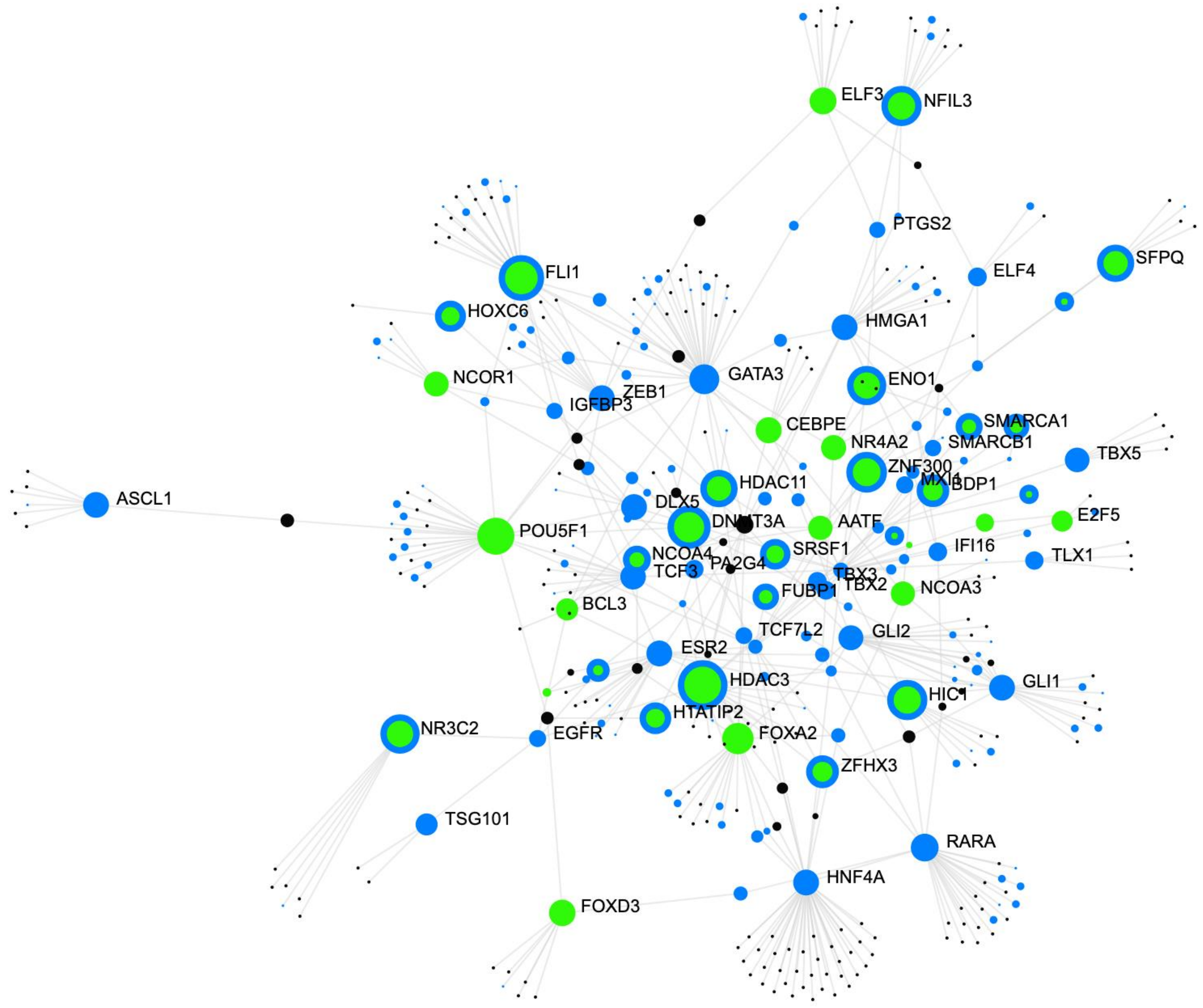

Figure S2: OmicsNet analysis of the transcription factors networks that can be generated using cluster 2 specific transcription factors. Large nodes with names refer to transcription factors, smaller nodes to target genes of the transcription factors. Blue color refers to transcription factors and target genes associated to “cell proliferation” GO Biological process term. In this figure are only represented TFs showing down-modulation in cluster 2 when compared with the other clusters.

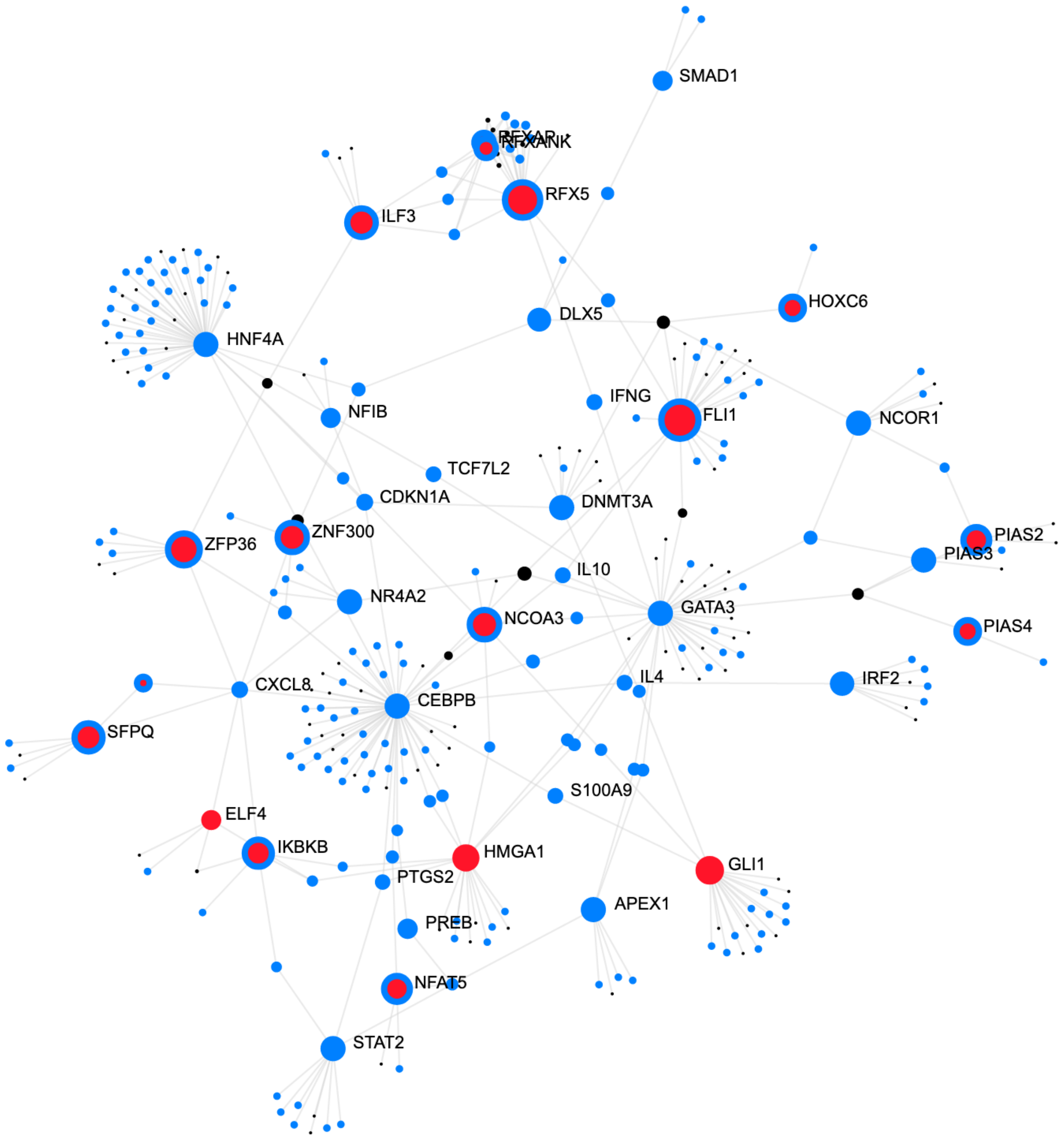

Figure S3: OmicsNet analysis of the transcription factors networks that can be generated using cluster 3 specific transcription factors. Large nodes with names refer to transcription factors, smaller nodes to target genes of the transcription factors. Blue color refers to transcription factors and target genes associated to "Chemical stimuli" GO Biological process term. In this figure are only represented TFs showing up-modulation in cluster 3 when compared with the other clusters.

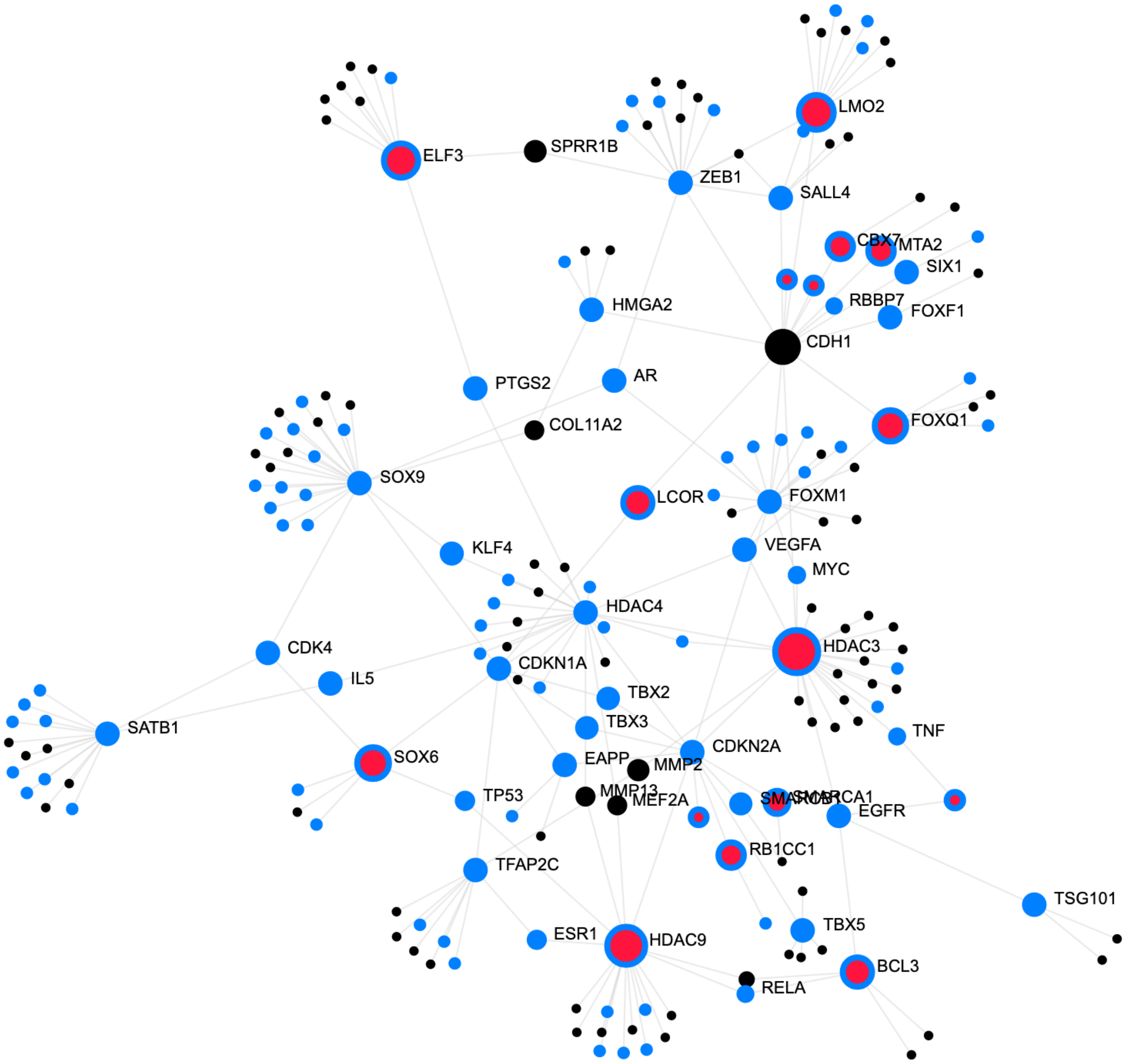

Figure S4: OmicsNet analysis of the transcription factors networks that can be generated using cluster 4 specific transcription factors. Large nodes with names refer to transcription factors, smaller nodes to target genes of the transcription factors. Blue color refers to transcription factors and target genes associated to "cell proliferation" GO Biological process term. In this figure are only represented TFs showing up-modulation in cluster 14when compared with the other clusters.

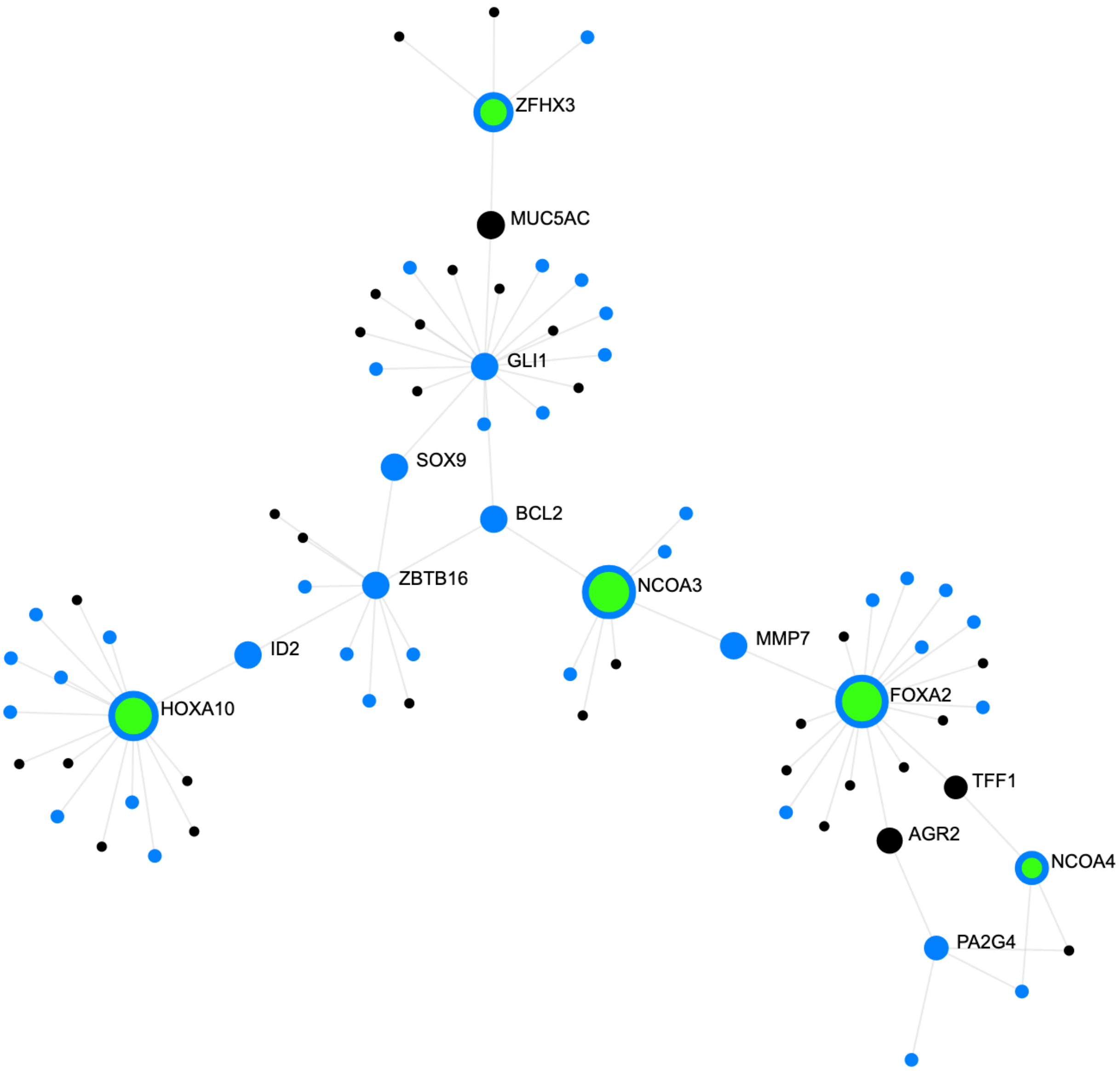

Figure S5: OmicsNet analysis of the transcription factors networks that can be generated using cluster 5 specific transcription factors. Large nodes with names refer to transcription factors, smaller nodes to target genes of the transcription factors. Blue color refers to transcription factors and target genes associated to “cell proliferation” GO Biological process term. In this figure are only represented TFs showing down-modulation in cluster 2 when compared with the other clusters.
